# Staffordshire Bull Terriers in the UK: their disorder predispositions and protections

**DOI:** 10.1186/s40575-020-00092-w

**Published:** 2020-09-23

**Authors:** Camilla Pegram, Katie Wonham, Dave C. Brodbelt, David B. Church, Dan G. O’Neill

**Affiliations:** 1 Pathobiology and Population Science, The Royal Veterinary College, Hawkshead Lane, North Mymms, Hatfield, Herts AL9 7TA UK; 2 The Donkey Sanctuary, Slade House, Devon, EX10 0NU UK; 3 Clinical Sciences and Services, The Royal Veterinary College, Hawkshead Lane, North Mymms, Hatfield, Herts AL9 7TA UK

**Keywords:** VetCompass, Electronic patient record, EPR, Breed, Dog, Epidemiology, Primary-care, Veterinary, Pedigree, Purebred, Staffordshire bull terrier, Staffie

## Abstract

**Background:**

The Staffordshire Bull Terrier is a popular dog breed in the UK but there is limited reliable evidence on disorder predispositions and protections within the breed. Using anonymised veterinary clinical data from the VetCompass™ Programme, this study aimed to identify common disorders with predisposition and protection in the Staffordshire Bull Terrier. The study hypothesised that Staffordshire Bull Terriers would have higher odds of aggression compared with non-Staffordshire Bull Terriers.

**Results:**

The clinical records of a random sample of dogs of all types were reviewed to extract the most definitive diagnoses for all disorders existing during 2016. A combined list from the 30 most common disorders in Staffordshire Bull Terriers and the 30 most common disorders in non-Staffordshire Bull Terriers was generated. Multivariable logistic regression was used to report the odds of each of these disorders in 1304 (5.8%) Staffordshire Bull Terriers compared with 21,029 (94.2%) non-Staffordshire Bull Terriers. After accounting for confounding, Staffordshire Bull Terriers had significantly increased odds of 4/36 (11.1%) disorders compared to non-Staffordshire Bull Terriers with highest odds for seizure disorder (OR 2.06; 95% CI 1.24 to 3.40; *p* = 0.005). Conversely, Staffordshire Bull Terriers had reduced odds of 5/36 (13.9%) disorders, with lowest odds for patellar luxation (OR 0.15; 95% CI 0.04 to 0.61; *p* = 0.008). There was no significant difference in the odds of aggression between Staffordshire Bull Terriers compared with non-Staffordshire Bull Terriers (OR 1.09; 95% CI 0.75 to 1.58; *p* = 0.644).

**Conclusions:**

This study provides a reliable evidence base of breed-specific disorder predispositions and protections that can be used by breeders to optimise breeding decisions. The findings can assist prospective owners of Staffordshire Bull Terriers to make informed decisions when acquiring a dog. From the relative number of predispositions to protections identified, there is no evidence that Staffordshire Bull Terriers have higher overall health problems than non-Staffordshire Bull Terriers.

## Plain English summary

The Staffordshire Bull Terrier (SBT) is currently a popular dog breed in the UK. However there is limited information on disorders to which SBTs are predisposed and disorders to which the breed is protected. Using veterinary clinical data from the VetCompass™ Programme at the Royal Veterinary College, this study aimed to identify disorders with predisposition and protection in the SBT. The study hypothesised that SBTs would have higher risk of aggression compared with non-SBTs.

The study included 1304 (5.8%) SBTs and 21,029 (94.2%) non-SBTs. SBTs were predisposed to 4/36 (11.1%) specific disorders compared to non-SBTs with the breed at over twice the risk of seizure disorder compared with dogs that were not SBTs. Conversely, SBTs were identified as protected to 5/36 (13.9%) specific disorders. Slipping kneecap (patellar luxation) had the lowest risk with SBTs having almost seven times less risk than dogs that were not SBTs. SBTs did not show increased risk of aggression compared with non-SBTs.

This study provides a reliable evidence base of breed-specific disorder predispositions and protections that can be used by breeders to optimise breeding decisions. The results also assist prospective SBT owners to make an informed decision when acquiring a dog. From the relative number of predispositions to protections identified, there is no evidence that SBTs have higher overall health problems than non-SBTs.

## Background

The Staffordshire Bull Terrier (SBT) was first recognised as a breed by the UK Kennel Club (KC) in 1935 [1]. Developed as a fighting dog in the UK in the nineteenth century, the breed is thought to have originated in Birmingham and Staffordshire by cross-breeding the bulldog and English terrier [1]. The SBT is currently a popular breed in the UK, recently identified as the second most common purebred in the wider general dog population under primary veterinary care [2]. Conversely, SBTs are less popular among the registered pedigree subset of UK dogs, being placed as the 12th most commonly registered breed by the KC in 2018–2019 [3], with registrations declining by 52.4% from 8663 in 2010 to 4124 in 2016 [4]. However, registrations have since remained relatively stable [4].

A recent textbook that reviewed the general literature for disease predispositions across all dog breeds reported SBTs as predisposed to 20 disorders, including aggression, atopic dermatitis, demodicosis, elbow dysplasia, mammary neoplasia and corneal ulceration. However these studies varied widely in study design, date, geographical location and comparator groups [5]. A genetic basis for aggression in SBTs has been described [6] and a survey of UK veterinarians classified SBTs with high aggression while SBTs were over-represented as the aggressor in dog-dog conflict in Germany [5–7]. SBTs are a common breed in rehoming centres and, due to their perceived behavioural issues, are reportedly difficult to rehome [8]. However, despite their reputation, given responsible ownership and an enriched environment, SBTs can make a suitable family pet [9]. Indeed, the UK KC Breed Standard describes SBTs as “highly intelligent and affectionate especially with children” [1].

The KC reports three inheritable conditions to which SBT’s are predisposed: hereditary cataracts (HC-HSF4), L-2-hydroxyglutaric aciduria (L-2HGA) and persistent hyperplastic primary vitreous (PHPV) [1]. Therefore, eye testing and DNA tests for HC-HSF4 and L-2HGA for SBT breeding parents are mandatory for KC assured breeders and eye screening for PHPV for all puppies is recommended [1]. However, despite the importance accorded to these conditions by these requirements, prevalence estimates for these conditions within the general UK population of SBTs are limited and the studies that do exist have reported these conditions as rare [10–13].

In order to better understand the relevance of individual disorders to the health profile of a breed, both absolute risk (prevalence) as well as relative risk information (predisposition and protection) need to be considered. Relatively small decreases in a highly prevalent disorder may confer substantial health advantage whereas even large reductions in a high predisposition for a rare disorder may offer minimal overall health impact for a breed [14]. A previous VetCompass™ study reported that certain purebreeds may have a higher prevalence of some common disorders compared with crossbred dogs. However, of the common, specific disorders, only obesity had a higher prevalence in SBTs compared to crossbred dogs (6.0% in SBTs vs. 3.9% in crossbred dogs). The prevalence estimates for other specific disorders were lower for SBTs compared to crossbreds, including: periodontal disease (2.4% vs 9.2%), degenerative joint disease (5.4% vs 7.5%), and lipoma (2.1% vs 3.8%), suggesting a relatively good overall health status for the breed [2].

Whilst SBTs are often considered relatively healthy [15], and with median longevity reported as 10.7–12.8 years [16, 17], evidence on disorder protection within SBTs, or indeed any breed, is limited. To date, breed protections to disorders have not been commonly reported within canine populations. However, deeper understanding of breed protections offers the potential to teach us as much about how to breed healthier dogs by breeding towards low disorder risk rather than just trying to breed away from high disorder risk as is the current preference in many dog breeding strategies [18].

Using anonymised veterinary clinical data from the VetCompass Programme [19], this study aimed to compare the odds of common disorders between SBTs and all remaining dogs under primary veterinary care in the UK during 2016 after accounting for major confounding variables and therefore to identify disorders with predisposition and protection in the SBT. The study hypothesized that SBTs have higher odds of aggression compared with non-SBTs. These results could assist breeders, veterinary practitioners and owners with an evidence base on the wider general population of dogs to predict, prevent and manage key health and welfare opportunities for SBTs.

## Methods

The study population included all available dogs under primary veterinary care at clinics participating in the VetCompass Programme during 2016. Dogs under veterinary care were defined as those with either a) at least one electronic patient record (EPR) (VeNom diagnosis term, free-text clinical note, treatment or bodyweight) recorded during 2016 or b) at least one EPR recorded during both 2015 and 2017. VetCompass collates de-identified EPR data from primary-care veterinary practices in the UK for epidemiological research [20]. Data fields available to VetCompass researchers include a unique animal identifier along with species, breed, date of birth, sex, neuter status, insurance status and bodyweight, and also clinical information from free-form text clinical notes, summary diagnosis terms [21] and treatment with relevant dates.

A cohort study design was used to estimate the one-year (2016) period prevalence of the most commonly diagnosed disorders in a random sample of SBTs and a random sample of all other dogs [22]. Sample size calculations for increased odds of aggression in SBTs in *Epi info (CDC)* estimated that approximately 681 SBTs and 13,610 non-SBTs would be needed to detect an odds ratio of ≥1.75, based on an estimated 5% of SBTs being recorded with aggression during the study period, with 80% power and 95% confidence assuming an approximate 20:1 ratio of non-SBTs to SBTs in the study population [2, 23]. Ethics approval was obtained from the RVC Ethics and Welfare Committee (reference number SR2018–1652).

Breed information entered by the participating practices was cleaned and mapped to a VetCompass breed list derived and extended from the VeNom Coding breed list [21]. Dogs recorded as SBT were categorised as SBT and dogs recorded with any other breed term were categorised as non-SBT. Neuter status was defined by the final available EPR neuter value and was combined with sex: female entire, female neutered, male entire and male neutered. Adult bodyweight was defined as the mean of all bodyweight (kg) values recorded for each dog after reaching 18 months old. Mean adult bodyweight was reported overall and broken down by sex for all breeds with adult bodyweight available for at least 100 dogs. Bodyweight was further categorized as “at or above the breed/sex mean”, “below the breed/sex mean” and “no recorded bodyweight”. Age (years) at the final study date (December 31, 2016) was categorised: ≤ 3.0, 3.0 to < 6.0, 6.0 to < 9.0, 9.0 to < 12.0 and ≥ 12.0. Veterinary group attended was categorised as 1–5, based on the 5 practice groups involved in the study. The practice groups included in the current study were distributed throughout the UK and were assigned a code during analysis to ensure anonymity. Insurance status was categorised as insured or not insured as recorded by the final available EPR.

The list of unique animal identification numbers for all dogs under veterinary care in 2016 was randomly ordered and the clinical records of a randomly selected subset of animals were reviewed manually in detail to extract the most definitive diagnoses recorded for all disorders that existed during 2016 [2]. Elective (e.g. neutering) or prophylactic (e.g. vaccination) clinical events were not included. No distinction was made between pre-existing and incident disorder presentations. Disorders described within the clinical notes using presenting sign terms (e.g. ‘vomiting’ or ‘vomiting and diarrhoea’), but without a formally recorded clinical diagnostic term, were included using the first sign listed (e.g. vomiting). Aggression was included as a specific disorder term and included all dogs where the clinical records showed evidence of aggressive behaviour of any type during 2016. This definition was applied equally between SBTs and non-SBTSs. The extracted diagnosis terms were mapped to a dual hierarchy of diagnostic precision for analysis: specific-level precision and grouped-level precision as previously described [2]. Briefly, specific-level precision terms described the original extracted terms at the maximal diagnostic precision recorded within the clinical notes (e.g. inflammatory bowel disease would remain as inflammatory bowel disease). Grouped-level precision terms mapped the original diagnosis terms to a general level of diagnostic precision (e.g. inflammatory bowel disease would map to gastro-intestinal).

Following data checking for internal validity and cleaning in Excel (Microsoft Office Excel 2013, Microsoft Corp.), analyses were conducted using SPSS version 24.0 (IBM Corp). The sex-neuter status, age, adult bodyweight and insurance status for SBTs and non-SBTs under veterinary care during 2016 were described.

One-year period prevalence values were reported separately for SBTs and non-SBTs to describe the probability of diagnosis at least once during 2016. The final combined list of 36 disorders included the 30 most common disorders in SBTs and the 30 most common disorders in non-SBTs. Continuous variables were non-normally distributed and so were summarised using median, interquartile range (IQR) and range. Mann-Whitney U test, chi-square test and Fisher’s exact test were used as appropriate for comparison of demographic data between cases and non-cases [24, 25]. Multivariable modelling using binary logistic regression was used to report the odds of each of these diseases in SBTs compared with non-SBTs. A separate model was created for each specific-level and grouped disorder. Information theory was applied to generate a list of confounding variables that was consistently included alongside the breed variable in each model [26, 27]. Breed was an a priori factor of interest and the models additionally included age (years), sex-neuter status, at/above or below mean bodyweight, insurance status and vet group. Model fit was assessed with the Hosmer-Lemeshow Test [28]. Statistical significance was set at the 5% level. All figures were created in R statistical software (R version 3.6.2) using the “forestplot” package [29].

## Results

The analysis included a random sample of 22,333 (2.5%) dogs from the study population of 905,544 dogs under veterinary care during 2016 in the UK. Of these, 1304 (5.8%) were SBTs, the second most common purebred breed. The other most common breeds included 1462 (6.5%) Labrador Retrievers, 1168 (5.2%) Jack Russell Terriers, 793 (3.6%) Shih-tzus, 771 (3.5%) Cocker Spaniels, along with 5981 (26.8%) crossbreeds. Data completeness were: breed 100.0%, age 98.8%, sex-neuter status 99.7%, insurance status 100.0% and bodyweight 66.6%.

Descriptive results were reported on 1304 SBTs and 21,029 non-SBTs (Table 1). The median age of SBTs (5.65 years, IQR 2.55–8.83, range 0.14–18.95) was older than for non-SBTs (4.33 years, IQR 1.84–7.98, range 0.01–20.46) (*p* <  0.001). The median bodyweight of SBTs (20.20 kg, IQR 17.68–22.86, range 12.47–31.00) was heavier than for non-SBTs of (12.65 kg, IQR 7.90–25.40, range 1.41–85.00) (*p* <  0.001).

**Table 1:** Descriptive statistics for demographic characteristics in SBTs (*n* = 1304) and non-SBTs (*n* = 21,029) under primary veterinary care in the UK

Variable	Category	SBT count (%)	Non-SBT count (%)	*P*-value
Age (years)	≤ 3	364 (28.2)	7776 (37.4)	< 0.001
	3 to < 6	332 (25.7)	5225 (25.1)	
	6 to < 9	288 (22.3)	3725 (17.9)	
	9 to < 12	195 (15.1)	2411 (11.6)	
	≥ 12	111 (8.6)	1639 (7.9)	
Sex-neuter status	Male entire	396 (30.5)	6081 (29.0)	< 0.001
	Male neutered	230 (17.7)	5011 (23.9)	
	Female entire	354 (27.3)	5330 (25.4)	
	Female neutered	318 (24.5)	4538 (21.7)	
At/above or below mean bodyweight for breed and sex	At or above	391 (30.0)	6437 (30.6)	0.264
	Below	451 (34.6)	7595 (36.1)	
	Not recorded	462 (35.4)	6997 (33.3)	
Insurance status	Insured	126 (9.7)	2853 (13.6)	< 0.001
	Not insured	1178 (90.3)	18,176 (86.4)	
Vet Group	1	10 (0.8)	67 (0.3)	0.075
	2	443 (34.0)	6903 (32.8)	
	3	60 (4.6)	945 (4.5)	
	4	223 (17.1)	3592 (17.1)	
	5	568 (43.6)	9522 (45.3)	

The *P*-value represents comparison of demographic variables between SBTs and non-SBTs

Of the SBTs, 831/1304 (63.7%) were diagnosed with ≥1 disorder compared with 13,873/21,029 (66.0%) of the non-SBTs. After using multivariable methods to account for effects of age, sex-neuter status, at/above or below mean bodyweight, insurance status and vet group, the odds of diagnosis with ≥1 disorder did not significantly differ in SBTs compared with non-SBTs (odds ratio [OR] 0.90; 95% confidence interval [CI] 0.80 to 1.02; *p* = 0.102).

**Fig. 1:**
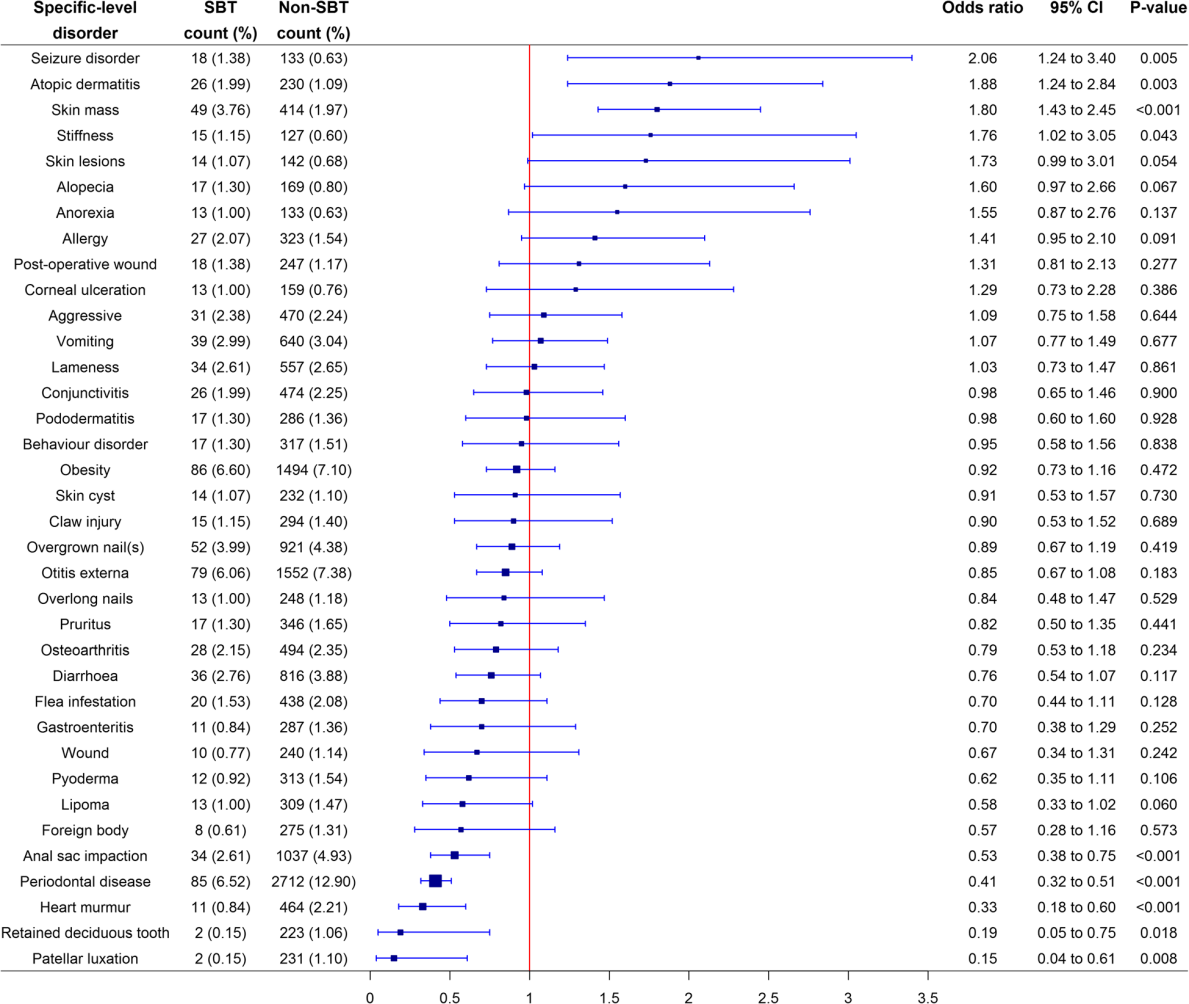
Forest plot of the multivariable logistic regression odds ratios with corresponding 95% CIs (confidence intervals) for the combined list from the 30 most common disorders in SBTs and in non-SBTs at a specific-level of diagnostic precision recorded in dogs under primary veterinary care at UK practices participating in the VetCompass™ Programme from January 1st 2016 to December 31st, 2016. Model variables accounted for included age, sex-neuter status, at/above or below mean bodyweight, insurance status and vet group. Specific-level precision describes the original extracted terms at the maximal diagnostic precision recorded within the clinical notes

At a specific-level of diagnostic precision, after accounting for confounding using multivariable methods, SBTs had significantly increased odds of 4/36 (11.1%) specific-level disorders compared to non-SBTs. These were: seizure disorder (OR 2.06; 95% CI 1.24 to 3.40; *p* = 0.005), atopic dermatitis (OR 1.88; 95% CI 1.24 to 2.84; *p* = 0.003), skin mass (OR 1.80; 95% CI 1.43 to 2.45; *p* < 0.001) and stiffness (OR 1.76; 95% CI 1.02 to 3.05; *p* = 0.043). Conversely, SBTs had significantly reduced odds of 5/36 (13.9%) specific-level disorders compared to non-SBTs. These were: anal sac impaction (OR 0.53; 95% CI 0.38 to 0.75; *p* < 0.001), periodontal disease (OR 0.41; 95% CI 0.32 to 0.51; *p* < 0.001), heart murmur (OR 0.33; 95% 0.18 to 0.60; *p* < 0.001), retained deciduous tooth (OR 0.19; 95% CI 0.05 to 0.75; *p* = 0.018) and patellar luxation (OR 0.15; 95% CI 0.04 TO 0.61; *p* = 0.008) (Fig. 1).

**Fig. 2:**
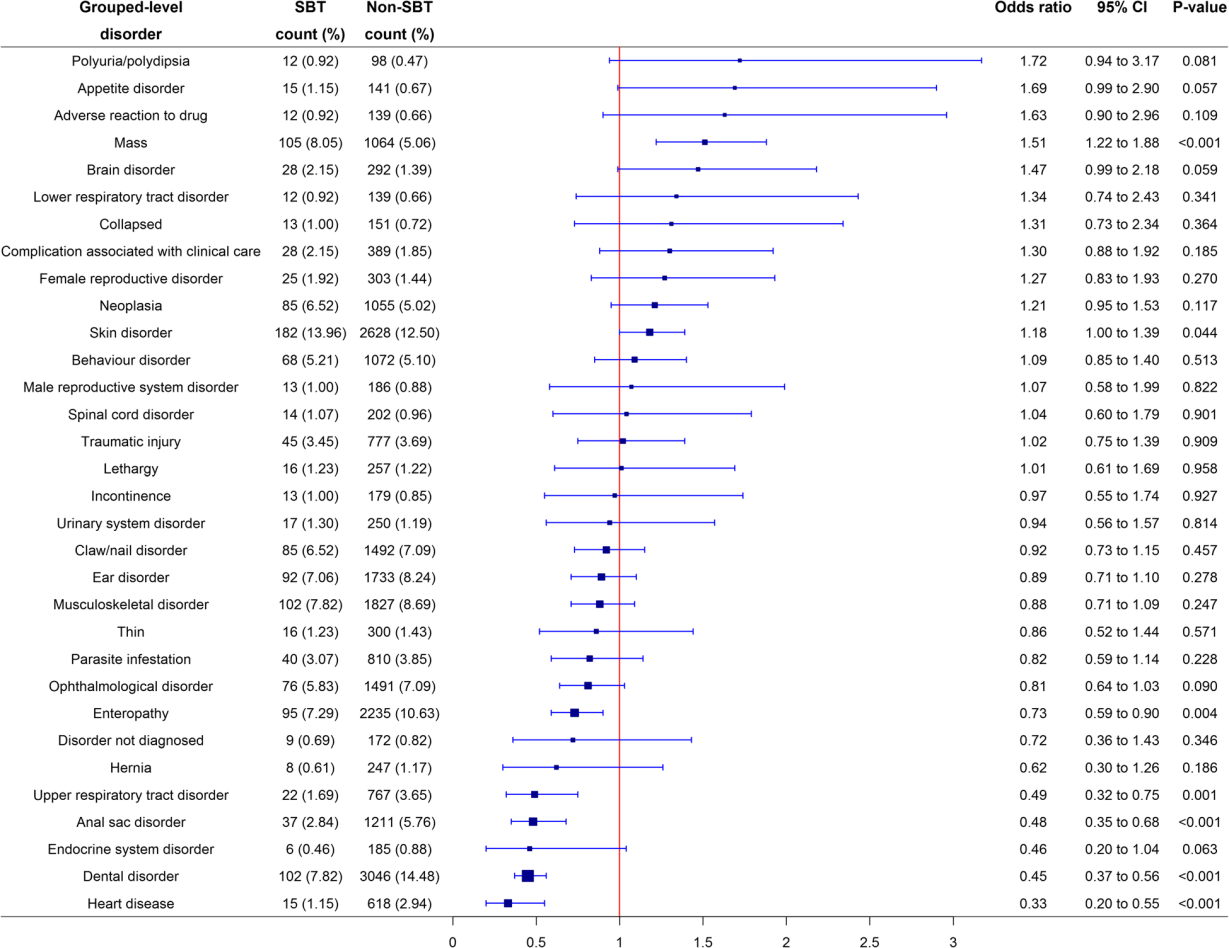
Forest plot of the multivariable logistic regression odds ratios with corresponding 95% CIs (confidence intervals) for the combined list from the 30 most common disorders in SBTs and in non-SBTs at a grouped-level of diagnostic precision recorded in dogs under primary veterinary care at UK practices participating in the VetCompass™ Programme from January 1st 2016 to December 31st, 2016. Model variables accounted for included age, sex-neuter status, at/above or below mean bodyweight, insurance status and vet group. Grouped-level precision describes the original extracted terms mapped to a general level of diagnostic precision

At a grouped-level of diagnostic precision, after accounting for confounding using multivariable methods, SBTs had significantly increased odds of 2/32 (6.3%) grouped-level disorders compared to non-SBTs. These were: mass (OR 1.51; 95% CI 1.22 to 1.88; *p* < 0.001) and skin disorder (OR 1.18; 95% CI 1.00 to 1.39; *p* = 0.044). Conversely, SBTs had reduced odds of 5/32 (15.6%) grouped-level disorders compared to non-SBTs. These were: enteropathy (OR 0.73; 95% CI 0.59 to 0.90; *p* = 0.004), upper respiratory tract disorder (OR 0.49; 95% CI 0.32 to 0.75; *p* = 0.001), anal sac disorder (OR 0.48; 95% CI 0.35 to 0.68; *p* < 0.001), dental disorder (OR 0.45; 95% CI 0.37 to 0.56; *p* < 0.001) and heart disease (OR 0.33; 95% CI 0.20 to 0.55; *p* < 0.001) (Fig. 2). The Hosmer-Lemeshow test indicated no evidence of poor model fit (*p* > 0.05) in any of these multivariable models.

## Discussion

This is the largest study to date using primary-care veterinary data that specifically aimed to report on SBT disorder predisposition and protection and used an underlying comparator group that reflected the entire remaining population of dogs under veterinary care. The study characterised the demography and health of a large cohort 22,333 dogs of which 1304 were SBTs under primary veterinary care in the UK. This has enabled reporting of relative predispositions to, and protections from, disorders, as well as absolute prevalence values using methods that have previously been restricted due to the inability to access the large data resources required for such analyses. This approach can now also be extended to explore the health of many other breeds.

SBTs were no more likely to have at least one disorder than non-SBTs (OR 0.90; *p* = 0.102). At a specific-level of diagnostic precision, SBTs had higher odds of 4/36 (11.1%) disorders compared to non-SBTs and had reduced odds of 5/36 (13.9%) disorders. There were 27/36 (75.0%) disorders with no significant difference in odds between SBTs and non-SBTs. At a grouped-level of diagnostic precision, SBTs had higher odds of 2/32 (6.3%) disorders and had reduced odds of 5/32 (15.6%) disorders compared to non-SBTs. There were 25/32 (78.1%) disorders with no significant difference in odds between SBTs and non-SBTs. Although severity and duration of disorder predispositions and protections should be taken into account when fully interpreting these results, these immediate results suggest no evidence that SBTs have higher overall health problems compared to the remainder of the general UK canine population. Despite this overall conclusion, the current study did identify some specific predispositions and protections that are very relevant to the breed.

The current study hypothesised that SBTs have higher odds of aggression compared with non-SBTs. This is an important question to answer because the perception of differential risk of aggression between SBTs and other breeds has a strong impact on the potential to rehome SBTs [8]. However, SBTs showed no significant difference in odds for aggression compared to non-SBTs (OR 1.09; *p* = 0.644). This contrasts with previous reports that SBTs showed higher levels of aggression compared to other dog breeds [6, 7], although many of these reported predispositions were documented over 20 years ago. It is possible there has since been a shift in breed behaviour, with more SBTs kept as family pets, rather than as a status symbol as they have been historically, reducing levels of aggression in the breed [9]. The current study findings suggest that potential owners visiting rehoming centres should avoid preconceptions about the breed behaviour, as given responsible ownership and an enriched environment SBTs can make a suitable family pet [9]. It should be noted that all forms of aggression were grouped and evaluated as a single disorder. The study design used for the current study based on a spectrum of disorders managed in primary-care practice precluded extraction of sub-categories of aggression with deeper context. However, future studies with specific focus on aggression could evaluate different forms of aggression within different dog breeds to gain a deeper understanding of these issues.

### Predispositions

SBTs showed predisposition to seizure disorder at a specific-level of diagnostic precision (OR 2.06), with prevalence of 1.38%. SBTs were previously overrepresented in a UK based study of 1260 epileptic dogs [30]. However, previous research based on primary-care data did not identify a significantly increased or decreased risk of epilepsy of unknown origin in SBTs compared with crossbreeds [31]. A further report with similar methodology identified SBTs at decreased risk (OR 0.72) of seizure disorder compared with Labrador Retrievers [32]. The contrasting findings in the current study may be due to the different case definitions and comparative populations used. The current study classified disorders according to their most precise diagnostic term, thus SBTs diagnosed with seizure disorder did not encompass those dogs with a more precise term, such as epilepsy. Therefore, the findings might suggest breed diagnostic differences rather than a true predisposition and should be interpreted with some caution. In addition, 18 SBTs were recorded with seizure disorder, therefore the relatively small number of cases identified may artificially inflate the odds ratio. SBTs have previously been reported as predisposed to L-2HGA, an inherited metabolic disorder which can result in seizures [10]. None of the 18 cases mentioned L-2HGA as a cause of seizure disorder, which may reflect the rarity of L-2HGA, however only 1/18 (5.6%) SBTs were tested based on the EPRs. There is no known treatment for L-2HGA and seizures are usually well controlled with anti-epileptic drugs [10]. Therefore, given that diagnosis of L-2HGA doesn’t necessarily alter treatment outcome, it might be that identification of this genetic mutation is a higher priority in SBTs used for breeding to reduce risk of affected offspring.

SBTs showed predisposition to atopic dermatitis at a specific-level of diagnostic precision (OR 1.88). This concurs with previous research that reported SBTs as the breed with the fifth-highest incidence of atopic dermatitis in insured dogs in Sweden (8.0 cases per 1000) [33]. Despite the predisposition identified, the prevalence of atopic dermatitis in the current study was relatively low (1.99%), compared with a US estimate of 8.7% in dogs overall [34]. Atopy is complex in its aetiology and diagnosis [35, 36] and therefore the true prevalence may be higher than reported here and thus the disorder should be considered important for the breed due to the associated negative impacts on quality of life [37]. At a grouped-level of diagnostic precision, SBTs showed predisposition to skin disorders (OR 1.18). Whilst this would include atopic dermatitis, and possible alternative diagnostic terms used for atopy, SBTs have previously been identified with predisposition to juvenile-onset demodicosis [38, 39]. Demodicosis did not feature within the top 30 specific disorders in SBTs or non-SBTs, however may have been included within skin disorders at a grouped-level.

SBTs showed predisposition to skin masses at a specific-level of diagnostic precision (OR 1.80) and masses at a grouped-level (OR 1.51), with prevalence of 3.76 and 8.05% respectively. A previous study based on UK primary-care data did not find a significant difference in skin mass prevalence between SBTs and crossbreeds, however confounding factors were not accounted for in this previous study and a different comparator group was used [2]. SBTs have documented predisposition to mast cell tumours (MCTs), which most commonly present as skin masses [40–43]. Although predisposition to neoplasia was not identified in the current study, it might be that a proportion of the skin masses were MCTs, but were not investigated further and so a definitive diagnosis not reached. SBTs have previously reported predispositions to gastric carcinoma [44], mammary carcinoma [45], and had the eighth-highest proportional mortality from neoplasia among pedigree breeds [17]. Therefore, it is possible that the predisposition to masses identified in SBTs, but not neoplasia, is reflective of diagnostic differences between breeds.

SBTs had 1.76 times the odds of stiffness at a specific-level of diagnostic precision compared with non-SBTs, with a prevalence of 1.15%. This finding should be interpreted with some caution as no significant predispositions were identified in similar categories, including lameness, osteoarthritis and musculoskeletal disorders. Stiffness is a clinical sign commonly associated with musculoskeletal disorders, but is not a formal biomedical diagnosis in itself [46]. Therefore, the predisposition to stiffness reported may be a reflection of the different language or diagnostic criteria used by veterinarians between different dog breeds. It may also indicate that musculoskeletal disorders in SBTs appear relatively mild, therefore “stiffness” may not be categorised at a further diagnostic level. Indeed, veterinarians rated Pitbull breed types with low pain sensitivity compared with other breeds and gave lower ratings for Pitbull breed types than the general public [47]. Given the prevalence of elbow dysplasia in SBTs previously reported as 31.3–33.3% [48, 49] and the documented stoicism of the breed [50], it might be that “stiffness” is indicative of an underlying condition that may warrant further investigation.

The UK KC offers eye testing and DNA tests for HC-HSF4 and L-2HGA in SBTs, inherited conditions to which the breed is reportedly predisposed [1]. These conditions were not identified in sufficient numbers to be included in the current study. This may reflect a true rarity of these disorders, or it could be that they are just not routinely tested for. Further studies evaluating the prevalence of these disorders would be useful to determine the value of genetic testing in the breed.

### Protections

SBTs showed protection to patella luxation at a specific-level of diagnostic precision (OR 0.15), with prevalence of 0.15%. This is in agreement with previous research based on primary-care data in which SBTs had 0.5 times the odds of patella luxation compared with crossbreeds [51]. As a protected breed, SBTs could be used as a model to explore conformational and other factors that may assist to develop strategies to reduce the prevalence in other higher-risk breeds, such as the Bichon and Bulldog.

SBTs showed protection to enteropathy (OR 0.78, prevalence 7.29%) and anal sac disorder (OR 0.48, prevalence 2.84%) at a grouped-level of diagnostic precision and anal sac impaction at a specific-level (OR 0.53, prevalence 2.61%). This is similar to previous research based on primary-care data in which prevalence of enteropathy and anal sac disorder were lower for SBTs compared with crossbreeds [2]. Although enteropathy is a broad term, soft faeces and inflammatory bowel disease have been associated with anal sac disease [52], therefore enteropathy and anal sac disorders are somewhat interlinked and may share some of the same risk factors, including diet [52, 53]. It might also be that reduced risk of anal sac disorders is linked to conformation, with SBTs being a highly muscular breed [1]. Further research exploring the mechanisms underlying these protective effects in SBTs may benefit the at-risk breeds in the population.

SBTs showed protection to dental disorders at a grouped-level of diagnostic precision (OR 0.45) and periodontal disease (OR 0.41) and retained deciduous tooth (OR 0.19) at a specific-level. This is in agreement with previous research based on a primary-care population in which SBTs had a lower prevalence of dental disorder compared to crossbreeds (3.0% versus 9.8%) [2]. Although SBTs had protection to dental disorders in the current study, they still had a relatively high prevalence of such conditions (7.82% for dental disorders and 6.52% for periodontal disease). Exploration of the reasons why SBTs are protected in comparison to other breeds may enable these protective factors to be increased so that the breed prevalence decreases further, and equally that the health and welfare of other breeds might also be improved.

SBTs showed protection to heart disease at a grouped-level of diagnostic precision (OR 0.33) and heart murmurs at a specific-level (OR 0.33), with prevalence of 1.15 and 0.84% respectively. This is in agreement with previous research in which prevalence of heart disease and heart murmur were lower for SBTs than for crossbreeds under primary-care in the UK [2]. In addition, SBTs have previously been reported at lower odds (OR 0.25) of degenerative mitral valve disease compared with crossbreeds [54]. It might be that the historic use of SBTs as a fighting dog and “muscular, active and agile” appearance [1] has resulted in residual protection to cardiac disorders even in today’s dogs. Therefore, this could suggest some value in using SBTs in cross breeding programmes to outcross other breeds affected with specific cardiac predispositions [55].

SBTs showed protection to upper respiratory tract disorder at a grouped-level of diagnostic precision (OR 0.49), with prevalence of 1.69%. To the authors’ knowledge this protection has not been documented previously, therefore future work may explore this novel finding in greater detail. SBTs have been reported as at-risk to Brachycephalic Obstructive Airway Syndrome (BOAS) [56, 57], which can be characterised by increased and/or abnormal upper respiratory tract noise [58]. BOAS was not one of the most common disorders recorded in SBTs or non-SBTs in the current study, however is thought to be underreported in veterinary clinic records due to psychological desensitisation via normalisation in commonly affected breeds [59]. SBTs have been documented as having “noisy breathing” [60], however it may be that this is “accepted” by owners and veterinary professionals as a typical breed characteristic rather than the possibility of associated underlying pathology. Given the popularity of brachycephalic (“flat-faced”) dogs, but their intrinsic health and welfare issues [61], breeding away from this phenotype in SBTs should be encouraged.

### Limitations

The limitations of this study include those reported in previous VetCompass publications based on similar methods that applied retrospective analysis of primary-care EPR data [2, 62]. Additionally, the current study did not account for differences in severity and duration between disorders, which could provide further insights into the nature and ranking of breed predispositions [63]. Comparing the relative number of predispositions to protections does not necessarily reflect breed health without a measure of severity, however the study findings highlight the types of conditions SBTs are predisposed to and protected from.

A number of the predispositions identified, including seizure disorder, skin mass, stiffness and mass and the protections, including heart murmur, were the residual groupings when a more precise diagnosis was not reached. Therefore, these findings need to be interpreted with some caution, as they may reflect diagnostic differences between SBTs and other dog breeds rather than a true predisposition or protection. The greatest confidence may be given to predispositions identified at the greatest level of diagnostic precision, namely atopic dermatitis.

Although dogs were classified using a dualist system as either ‘SBT’ or ‘non-SBT’, the high proportion (26.8%) of crossbreeds among the non-SBTs might have caused some dilution of the phenotype given that some of these crossbreds may have included some SBT parentage. In addition, SBTs can be difficult to identify based on appearance alone, with some disagreement in classification of SBTs and Pit Bulls (based on photographs) reported between UK and US shelter workers [64]. However, difficulty in breed classification is not necessarily confined to one specific breed type, therefore it is unlikely misclassification was unidirectional.

This study used multiple comparisons. Strict adherence to a cut-off *P-value* of 0.05 to infer significance for multiple comparisons can lead to a Type 1 error of accepting false positive results. Furthermore, the small number of cases in some disorders reported might have led to a type 2 error of accepting false negative results [65]. We recommend that readers do not rely on the *P-values* of odds ratios alone, but consider the confidence intervals and prevalence percentages when interpreting the current results [66]. Consequently, the individual results for each of the disorders assessed should be interpreted as exploratory rather than confirmatory.

## Conclusion

This study evaluated a range of disorders and should be considered exploratory rather than confirmatory. The individual results should be confirmed in future a priori studies to increase confidence in the findings. From the relative number of predispositions to protections identified, there is no evidence that SBTs have higher overall health problems than non-SBTs. The study hypothesized that SBTs would have higher odds of aggression than non-SBTs, however no significant difference in odds was identified. The type of disorder predispositions and protections reported suggest diagnostic differences between dog breeds, which further work might help elucidate.

## Data Availability

The datasets generated during and/or analysed during the current study will be made available at the RVC Research Online repository (http://researchonline.rvc.ac.uk/id/eprint/12715).

## Abbreviations

CI: Confidence interval
EPR: Electronic patient record
HC-HSF4: Hereditary cataracts
IQR: Interquartile range
KC: The Kennel Club
L-2HGA: L-2-hydroxyglutaric aciduria
OR: Odds ratio
PHPV: Persistent hyperplastic primary vitreous
SBT: Staffordshire Bull Terrier
